# Regulatory developments in the conduct of clinical trials in India

**DOI:** 10.1017/gheg.2015.5

**Published:** 2016-02-23

**Authors:** R. Roy Chaudhury, D. Mehta

**Affiliations:** 1 Task Force for Research, Apollo Hospitals Educational and Research Foundation (AHERF), New Delhi, India; 2 Health and Environmental Law, Vidhi Centre for Legal Policy, New Delhi, India

**Keywords:** Central drugs standard control organisation, clinical trials, confidence-building measures, Indian regulatory framework

## Abstract

There has been a drop in clinical research in India following stringent conditions put in place by the Indian Supreme Court in 2013. The Court's orders came in the wake of irregularities highlighted in the conduct of clinical trials in the country. This paper highlights the steps taken by the Indian regulator, the Central Drugs Standard Control Organisation to comply with these directions. These are of three kinds: strengthening regulatory institutions, protecting participant safety and creating regulatory certainty for sponsors and investigators. Examples include the large-scale training of Ethics Committees, framing detailed guidelines on compensation and audiovisual recording of the informed consent process, as well as reducing the time taken to process applications. It is expected that these measures will inspire confidence for the much-needed resumption of clinical research.

## Introduction

In September 2013, the Supreme Court of India (SC) recommended more stringent controls on the conduct of clinical trials in order to protect the rights of participants in such trials [1]. These orders were passed in response to public interest litigation (PIL) filed by the *Swasthya Adhikar Manch* (Health Rights Forum), a non-governmental organisation that had documented irregularities and unethical practices in clinical trials conducted in the state of Madhya Pradesh. These included enrolling participants without obtaining proper informed consent and failing to compensate participants adequately for trial-related injuries or deaths. The PIL also asked for an investigation of approximately 400 trials that had been carried out in the 5 years between 2007 and 2012. In response to the SC's observations and directions, the Indian regulator, the Central Drugs Standard Control Organisation (CDSCO) introduced a slew of measures, some of which have been blamed for a significant drop in the number of clinical trials being conducted in India, with both domestic and foreign drug companies moving to alternate clinical trial sites [2, 3].

Developments in the SC coincided with the tabling, in May 2012, of the 59^th^ Report of the Parliamentary Standing Committee on Health and Family Welfare on the functioning of CDSCO. This report also made scathing comments on the regulation of clinical trials in India, providing examples of conflict of interest and a lack of transparency. It recommended urgent restructuring of the drug regulatory system in the country. In response to this report, the Ministry of Health and Family Welfare, in July 2013, constituted an Expert Committee headed by Professor Ranjit Roy Chaudhury (one of the authors of this article) to review the existing system and make recommendations to improve and strengthen it.

It is the aim of this paper to highlight the measures undertaken by CDSCO since 2013 to give effect to the recommendations of the Ranjit Roy Chaudhury Committee (RRC Committee) as well as the directions of the SC, to discuss the manner in which these have been implemented, and to point out what steps remain to be taken. These measures are discussed under three broad headings. The first is concerned with strengthening the institutions that are involved in reviewing and approving clinical trials, the second deals with steps taken specifically to protect the rights of clinical trial participants, while the third deals with measures that CDSCO has taken to reduce uncertainty and delay for clinical trial sponsors and investigators.

## Strengthening the institutional architecture

One of the biggest failings of the regulatory system in India so far has been the lack of sufficient trained personnel capable of effectively reviewing and monitoring clinical trials, including adequately robust Ethics Committees. Recognising this deficit, the report of the RRC Committee (RRC Report) made recommendations to improve the quality of the review process. These recommendations and the manner in which they have been implemented are set out below.

### Review by CDSCO

#### Recommendation

The RRC Report recommended the creation of a single Technical Review Committee (TRC) to replace the 12 new drug advisory committees that currently review applications. This committee was to be assisted by subject-specific experts to be selected from a Roster of Experts that would be set up after a nationwide search.

#### Implementation

Although a single body has not yet replaced the 12 different committees, the latter have instead been constituted into Subject Expert Committees (SECs) [4], with the members for each meeting to be drawn randomly from a pool of experts. CDSCO has also passed an order approving 25 panels of experts in various therapeutic areas [5]. In addition to these expanded SECs, a High-Powered Expert Committee, also known as the Technical Committee, has been set up to meet monthly and review the recommendations of the SECs. These are then approved, rejected or sent back to the SECs with modifications. Together, these committees have ensured that a high degree of scientific rigour is now brought to the review process, and detailed reasons are provided while approving or rejecting applications for clinical trials, in accordance with the directions of the SC.

This reorganisation has also considerably speeded up the approval process. Under the earlier setup, decisions were often stalled because committees had not met in over a year, or even if one or two members of the committee were absent [6]. However, the new processes put in place by CDSCO have ensured that applications are speedily, yet competently reviewed, bringing the pendency level down to zero [7]. It will be interesting to observe whether the efficiency of the new system can be maintained when the volume of clinical trial applications increases.

### Accreditation

#### Recommendation

The RRC Report recommended the accreditation of the different stakeholders involved in clinical trials, including principal investigators, trial sites and Ethics Committees. The report also stated that a Central Accreditation Council ought to be set up to put in place this accreditation.

#### Implementation

Even before these recommendations were published, the Drugs and Cosmetics Rules, 1945 (the Rules) had been amended in 2013 requiring Ethics Committees to obtain registration from the Licensing Authority before they could be permitted to review clinical trial protocols [8]. To bring this rule into effect, CDSCO has put in place a system for the pre-screening of applications for registration by Ethics Committees. A checklist of documents required to be submitted by these committees has also been made available.

Commendable, large-scale efforts have been made to ensure rigorous training for the members of Ethics Committees. Over 800 members have undergone training through programmes conducted by two organisations – the Clinical Development Services Agency, a unit of the Department of Biotechnology and CREATE, a consortium of the All India Institute of Medical Sciences, Christian Medical College, Vellore and the Forum for Ethics Review Committees in India.

Simultaneously, steps have been taken to put in place a system for the accreditation of clinical trial centres and principal investigators. The Quality Council of India, an autonomous body that was set up to introduce National Accreditation Programmes, has been entrusted with this responsibility and is currently in the process of reviewing Standard Operating Procedures and developing standards for quality assurance during the accreditation of investigators, trial sites and Ethics Committees. Ethics Committees will be accredited in the first phase, followed by clinical trial centres and Ethics Committees. The newly created Department of Health Research will be a partner in this massive exercise. Accreditation will provide a sense of confidence that the trials will be carried out ethically and that participants will be protected.

However, in order to have teeth, the accreditation process must eventually be accompanied by a legal prohibition on conducting trials at non-accredited centres and through non-accredited investigators.

## Protecting clinical trial participants

The most important recommendations of the RRC Report naturally deal with safeguarding the rights of participants through all the stages of a clinical trial, beginning with determining whether the trial needs to be conducted in the first place, right up to ensuring that appropriate compensation is awarded for trial-related injuries or death. Many of these recommendations have been incorporated almost verbatim in a series of orders passed by CDSCO on 3 July 2014. The steps taken by CDSCO to operationalise these recommendations are set out below.

### Compensation

#### Recommendation

The legal provisions on compensation have created much uncertainty because of frequent amendments, posing problems for participants and sponsors alike. The RRC Report recommended that compensation be related to causality, that it need not be provided for the therapeutic inefficiency of an investigational product and that strong provisions be made for providing ancillary care for other illnesses during the trial.

#### Implementation

The CDSCO has clarified that compensation is to be provided to participants or their nominees even in cases of injury or death discerned at a later stage, so long as such injury or death is determined to be trial-related [9]. The amended Rules also state that compensation will be payable for failure of the investigational product to provide the intended therapeutic effect or for the use of placebo in a placebo-controlled trial only where the standard care, although available, was not provided according to the clinical trial protocol. Although the intent of this amendment was to reduce the open-ended financial liability of sponsors under the earlier version of the Rules [10], clarification regarding the definition of ‘standard care’ is still required to ensure complete certainty regarding the obligations of sponsors.

An office order passed in July 2014 also requires sponsors to provide ‘ancillary care’ to trial participants suffering from any other illness during the trial [11]. Again, this phrase must be defined more clearly in order to ensure that sponsors are not required to bear the expenses of entirely unrelated illnesses suffered by participants during the trial.

CDSCO has also set up Expert Committees to frame formulae that will be applied to determine compensation, both for death as well as for different categories of injuries such as permanent disabilities, congenital anomalies and life-threatening illnesses. These formulae have now officially been recommended through a CDSCO order passed in December 2014 [12]. The incorporation of this level of detail in a legally binding instrument is a unique step, given that other jurisdictions usually only lay down a broad obligation to compensate, leaving further guidance about the method of payment and quantum of money to be evolved by industry [13].

### Demonstrating the necessity of clinical trials

One of the primary concerns regarding the conduct of clinical trials expressed in the 59^th^ Report as well as by the SC related to the treatment of Indian patients as human guinea pigs by multinational drug companies. The RRC report, as well as the SC, have made the following recommendations to ensure that trials are conducted in a way that is sensitive to the needs of the local population.

#### Recommendation

The RRC Report observed that ethnic differences can affect the efficacy, safety and dose regimen of a medicine. It recommended that certain factors such as the choice of control group and regional medical practice be taken into consideration while determining whether available data regarding a drug is ethically sensitive or not.

#### Implementation

CDSCO passed an order requiring the ethnicity of the local population to be factored in while granting approvals for new drugs. Thus, SECs must take into account certain properties of compounds that are likely to make them more sensitive to ethnic factors [14].

#### Recommendation

The RRC Report recommended that only drugs that fulfil a real medical need ought to be made available. In October 2013, the SC also attached conditions to approvals of clinical trials by requiring the following three factors to be determined in relation to new drugs – risk/benefit assessment, the innovativeness of the new drug *v*. the existing therapeutic option, and unmet medical need in the country.

#### Implementation

The three factors in the SC order have now been incorporated in a draft amendment to the Rules published in the Official Gazette in February 2015 [15]. This amendment makes an addition to Form 44 as well as Appendix I of Schedule Y, requiring the entity seeking permission to conduct clinical trials to submit data regarding these three factors. Decisions of the TRC granting approvals for clinical trials now also justify the manner in which these three criteria are met, and are publicly accessible on the CDSCO website.

Another measure that has been taken to protect the rights of participants is an order stating that placebo-controlled trials (which have also been under the scanner) will be permitted only when the trial is designed in an ‘appropriate, efficient and ethical’ way [16]. However, official guidance on how these parameters are supposed to be met remains to be worked out.

Thus, measures for the protection of participants have been implemented without imposing onerous obligations on sponsors and investigators. The actions taken by CDSCO to encourage the conduct of clinical research in India are discussed in the next section.

## Confidence building measures for investigators and sponsors

The regulatory system has been criticised for undue delay in processing applications, as well as for the unclear manner in which the obligations of sponsors and investigators are defined. The following measures have since been taken by CDSCO to assuage some of these concerns.

### Application process

#### Recommendation

The RRC Report stated that the hallmark of a good regulatory agency is a ‘transparent, time-bound decision-making process with clear-cut timelines.’ It recommended that deadlines be set for different activities and a transparent website be maintained with up-to-date information.

#### Implementation

First, CDSCO has set itself targeted timelines within which to process applications, with 6 months being the outer limit for the review of applications for the approval of new drugs and clinical trials. As already mentioned earlier, CDSCO has cleared its entire backlog, and in a bid to become more accessible to applicants, it arranges for daily public grievance meetings at the office of the Drugs Controller General of India (DCGI). Additionally, it proposes to introduce a system of formal Pre-Submission meetings that will be arranged between applicants, CDSCO officers as well as subject experts. These meetings will allow regulatory pathways to be tailored for particular applicants depending upon the information submitted by them. The idea behind this system is to introduce ‘transparency, accountability, predictability and speedy disposal’ [17]. Steps are also being taken to ensure the use of information technology at every stage of the clinical trial process and in September 2015, the CDSCO launched a new online submission system for clinical trials applications.

### Obligations relating to medical management

Sponsors have expressed uneasiness in the past regarding the open-ended provisions for compensation under the Rules. In particular, they have objected to rules that would appear to require them to provide free medical treatment to participants, irrespective of whether or not the injury was trial-related.

#### Recommendation

The RRC Report recommends that compensation need not be paid for death or injury due to ‘totally unproven unrelated causes.’ However, if an Adverse Event or Serious Adverse Event occurs during a clinical trial, the sponsor is to be responsible for providing medical care and treatment to the patient at his cost till the resolution of the event.

#### Implementation

Sub-rule (1) of Rule 122DAB of the previous version of the Rules required the trial participant to be provided with ‘free medical management as long as required’. An amendment to the rules in December 2014 restricts the scope of this obligation to provide that the liability of the sponsor ends once it is established that the injury is not related to the clinical trial. However, Expert Committees setup to determine this causality must also function quickly and efficiently in order to ensure that sponsors are not required to bear the expenses of medical treatment for injuries that may prove to be unrelated to the trial.

### Audiovisual recording of the informed consent process

#### Recommendation

The RRC Report recommended that the audiovisual recording of the informed consent process be undertaken in the case of research on vulnerable populations that have a diminished capacity to protect their own interests. Audiovisual recording was also recommended for those participants that were willing to participate, but did not wish to provide written consent.

#### Implementation

CDSCO went a step further than the recommendation in the RRC Report and required audiovisual recording of the informed consent process to be made mandatory for all participants in clinical trials, vulnerable or otherwise [18]. This order was met with protest from the clinical trial industry and has now been modified through an amendment to the Rules in July 2015 [19]. The amendment restricts the audiovisual recording of the informed consent process to vulnerable subjects in clinical trials involving ‘new chemical entities’ or ‘new molecular entities’. It also states that only audio recording will be needed in clinical trials involving anti-HIV and anti-leprosy drugs.

Thus, the amendment is now in conformity with the original recommendations made by the RRC Committee. This readiness on the part of the regulator to modify its decisions speaks well of its flexibility and willingness to change.

This flexibility is also exhibited in the decisions taken by the CDSCO to reverse the original restrictions that it had placed on the number of trials that one investigator could undertake at a time, as well as the minimum number of beds that were required for a hospital to be eligible as a clinical trial centre. These restrictions have now been lifted, and it is the Ethics Committees that have been entrusted with determining the suitability of clinical trial sites as well as the capacity of the investigator to undertake more than three trials at a time [20].

This willingness of the regulator to engage with civil society and industry is an encouraging start towards the creation of a conducive climate for clinical research in the country.

## The way forward

CDSCO has exerted itself remarkably in implementing the orders of the SC to ensure that clinical trials adhere to the strongest ethical practices. However, some of these measures may have been over-zealous and have had the effect of discouraging clinical research. Nevertheless, CDSCO has shown refreshing signs of its willingness to revisit these measures, and greater awareness about these changes must now be raised among investigators, industry and the general public. Steps must also be taken to regularly monitor the implementation of the RRC recommendations by CDSCO.

From a legal perspective, the steps that CDSCO has taken have been accomplished primarily through executive orders and administrative instructions. These may carry greater weight when consolidated and re-enacted in the form of primary legislation and revised rules. Similarly, ambiguity in the manner in which the rules relating to the provision of medical management and compensation have been drafted requires clarification. Providing sponsors and investigators the opportunity to make representations during causation assessments by the Expert Committees ought also to be considered. Greater attention to legal drafting will go a long way in inspiring confidence about the scope and content of CDSCO's orders.

There are other gaps that need to be filled by CDSCO in order to operationalise its various orders. For example, at a round table meeting of representatives from the clinical trial industry in May 2015, it was agreed that an inspection check-list ought to be created for SECs to allow them to review clinical trial protocols effectively, keeping in mind the additional criteria on risk–benefit assessment, innovativeness and unmet medical need laid down by the SC. There is also need for more uniform functioning and decision making by the members of the SECs. Industry has additionally emphasised the need to create detailed Standard Operating Procedures for Pre-Submission Meetings. Continuous engagement with relevant stakeholders is vital in ensuring that realistic and workable measures are adopted.

The draft Drugs and Cosmetics (Amendment) Bill 2015 amends the existing Drugs and Cosmetics Act, 1940, to include a separate chapter on clinical trials for the first time. The Bill also proposes to imposes stringent criminal penalties on sponsors and investigators for violating the conditions of permission of a clinical trial. The manner in which the Bill is drafted does not permit exceptions to be made for mistakes made by investigators acting in good faith. Such penalties are likely to have a severe chilling effect on the conduct of criminal trials in the country. The provisions of this Bill need serious reworking if they are not to have an even more detrimental effect on clinical research in India.

CDSCO has made a good start towards addressing the deficiencies in the existing regulatory framework. This start needs to be built on more constructively through a combination of measures: extensive capacity building and training, and thoughtful regulation and legislation. These steps will help bring India back on the path towards becoming a global clinical trial hub while ensuring that the highest ethical standards are maintained.

## Conclusions

The changes already made have been well received by the pharmaceutical industry, academia and civil society. This itself is a remarkable achievement. Several measures being implemented are being done probably for the first time in a country of this size with one thousand clinical trial centres. These include the detailed accreditation process, the formula for calculating compensation and an immense programme of training of members of Ethics Committees. The CDSCO has been strengthened already with 15 Assistant Drug Controller and 148 Drug Inspectors and more posts have been created and filled up. The total management of the system will be based on information technology. The number of clinical trials being initiated in the country is already increasing. India is set on a path to carry out transparent and robust, clinical trials based on ethics and scientific principles.
